# Committee on Exhibits, World's Columbian Dental Congress

**Published:** 1893-04

**Authors:** 


COMMITTEE ON EXHIBITS, WORLD'S COLUMBIAN DENTAL CONGRESS.—The committee on exhibits for the World's Columbian Dental Congress desires to obtain rare specimens of growths, abnormalities, casts, illustrations of methods, instruments and appliances, both ancient and modern, whereby the growth of the profession may be shown from its early fancy up to the present time. They also desire to exhibit an ideal library, operating room and laboratory; and to this end, earnestly requests all members of the profession, together with dental dealers and publishers, to loan them any specimens, instruments, appliances, books, photographs or pictures of societies and eminent men, of all countries, together with anything and everythiug that will be of interest to any dentist from any part of the world. They will pay all transportation charges on such exhibits to Chicago and return, and will insure the same, while on exhibition, if desired.

*Committee.*—Chas. P. Pruyn, chairman, 70 Dearborn street, Chicago, Ill.; Arthur E. Matteson, 3700 Cottage Grove avenue, Chicago, Ill.; E. M. S. Fernandes, 36 Washington street, Chicago, Ill.; M. L. Rhein, 104 E. Fifty-eight street, New York; A. W. McCandless, 1001 Masonic Temple, Chicago, Ill.; R. C. Young, Anniston, Ala.; James Chace, Ocala, Fla.; W. A. Campbell, Gold and Fulton streets, Brooklyn, N. Y.

Address all communications to Dr. A. W. McCandless, Secretary, 1001 Masonic Temple, Chicago, Ill.

